# Supplementary material for: A Case Study of the Morphological and Molecular Variation within a Ciliate Genus: Taxonomic Descriptions of Three Dysteria Species (Ciliophora, Cyrtophoria), with the Establishment of a New Species
Source: Int J Mol Sci. 2022 Feb 3;23(3):1764. doi: 10.3390/ijms23031764 (PMC8836684; doi:10.3390/ijms23031764)
Supplement: Supplementary file 1 [file ijms-23-01764-s001.zip › Table S1(2).pdf]

Table S1. Morphological comparison of populations of *Dysteria crassipes*.

Characters	Original population	Yellow Sea	Bohai Sea	South China Sea	East China Sea
Body length in vivo	–	50–80	40–60	50–60	50–65
Body shape	Rectangular to pentagonal	Rectangular to pentagonal	Rectangular	Rectangular	Rectangular
No. of RK	4	4	4	4	4
No. of FVK	2	2	2	2	2
No. of LK	–	5–9	7–8	6–8	5–9
No. of basal bodies in FvK	–	156–186	74–106	89–121	93–131
No. of basal bodies in TF	–	7–10	5–6	6–7	3–7
No. of basal bodies in EF	–	9–34	3–17	4–20	5–20
Data source	[31]	[8]	[9]	[5]	Present work

EF, equatorial fragment; FvK, frontoventral kineties; LK, left equatorial kineties; RK, right kineties; SK, somatic kineties; TF, terminal fragment.
–Data not available
