# Supplementary material for: A Case Study of the Morphological and Molecular Variation within a Ciliate Genus: Taxonomic Descriptions of Three Dysteria Species (Ciliophora, Cyrtophoria), with the Establishment of a New Species
Source: Int J Mol Sci. 2022 Feb 3;23(3):1764. doi: 10.3390/ijms23031764 (PMC8836684; doi:10.3390/ijms23031764)
Supplement: Supplementary file 1 [file ijms-23-01764-s001.zip › Table S2(2).pdf]

Table S2. Morphological comparison of populations of *Dysteria brasiliensis*.

Characters	Rio de Janeiro	Yellow Sea	Yellow Sea	Bohai Sea	South China Sea	Haikou	East China Sea Ningbo population-I	East China Sea Ningbo population-II	East China Sea Ningbo population-IV	East China Sea Ningbo population-III	East China Sea Ningbo population-V
Body length in vivo	150	100–130	55–140	85–125	115–175	85–135	105–125	115–135	115–120	170–210	120–160
Body shape	Triangular	Triangular or elongate, with a dorsal spine	Roughly triangular, with a dorsal spine	Triangular or elongate, With a dorsal spine	Triangular or elongate, with a dorsal spine	Elongate trapezoid with a dorsal spine	Triangular or elongate, With a dorsal spine	Triangular or elongate, With a dorsal spine	slim and curved	slim body with a prominent dorsal spine	Triangular or elongate, With a dorsal spine
No. of RK	–	5	5	5	5	5	5	5	5–6	5	5
No. of FVK	–	2	2	2	2	2	2	2	2	2	2
No. of LK	–	7-8	–	7–8	7-8	7–8	2-9	4–8	5–8	6–8	4–10
No. of basal bodies in FvK	–	–	–	190–250	220–300	–	182–283	206–293	186–271	188–353	189–261

No. of basal bodies in TF	–	13–15[b]	–	7–12	12–14	8–17	11–17	9-17	7–16	10–18	9–15
No. of basal bodies in EF	–	14–30[b]	–	8–23	11–28	6–34	8–33	7–26	8–23	9–30	7–25
Data source	[32]	[33]	[8]	[9]	[9]	[11]	Present work	Present work	Present work	Present work	Present work

EF, equatorial fragment; FvK, frontoventral kineties; LK, left equatorial kineties; RK, right kineties; SK, somatic kineties; TF, terminal fragment.
–Data not available. [b]Counted from illustration
